# How Diet Intervention via Modulation of DNA Damage Response through MicroRNAs May Have an Effect on Cancer Prevention and Aging, an *in Silico* Study

**DOI:** 10.3390/ijms17050752

**Published:** 2016-05-19

**Authors:** Felicia Carotenuto, Maria C. Albertini, Dario Coletti, Alessandra Vilmercati, Luigi Campanella, Zbigniew Darzynkiewicz, Laura Teodori

**Affiliations:** 1 Diagnostics and Metrology (FSN-TECFIS-DIM), ENEA, C.R. Frascati, Rome 00044, Italy; carotenuto@med.uniroma2.it (F.C.); vilmercati.ale@tiscali.it (A.V.); 2 Department Clinical Sciences and Translational Medicine, University ofRome Tor Vergata, Rome 00133, Italy; 3 Department of Biomolecular Sciences, University of Urbino “Carlo Bo”, Urbino 61029, Italy; maria.albertini@uniurb.it; 4 Department of Biological Adaptation and Aging B2A, University Pierre et Marie Curie Paris 06, Paris 75252, France; dario.coletti@upmc.fr; 5 Fondazione San Raffaele, Ceglie Messapica, Brindisi 72013, Italy; 6 Department of Chemistry, University of Rome “Sapienza”, Rome, 00185, Italy; luigi.campanella@uniroma1.it; 7 Department of Pathology, New York Medical College, Valhalla, NY 10595, USA; z_darzynkiewicz@nymc.edu

**Keywords:** food, bioactive compounds, n3-PUFA, resveratrol, curcumin, epi-gallocatechin-3gallate, chemoprevention

## Abstract

The DNA damage response (DDR) is a molecular mechanism that cells have evolved to sense DNA damage (DD) to promote DNA repair, or to lead to apoptosis, or cellular senescence if the damage is too extensive. Recent evidence indicates that microRNAs (miRs) play a critical role in the regulation of DDR. Dietary bioactive compounds through miRs may affect activity of numerous genes. Among the most studied bioactive compounds modulating expression of miRs are epi-gallocatechin-3-gallate, curcumin, resveratrol and n3-polyunsaturated fatty acids. To compare the impact of these dietary compounds on DD/DDR network modulation, we performed a literature search and an *in* *silico* analysis by the DIANA-mirPathv3 software. The *in silico* analysis allowed us to identify pathways shared by different miRs involved in DD/DDR *vis-à-vis* the specific compounds. The results demonstrate that certain miRs (e.g., -146, -21) play a central role in the interplay among DD/DDR and the bioactive compounds. Furthermore, some specific pathways, such as “fatty acids biosynthesis/metabolism”, “extracellular matrix-receptor interaction” and “signaling regulating the pluripotency of stem cells”, appear to be targeted by most miRs affected by the studied compounds. Since DD/DDR and these pathways are strongly related to aging and carcinogenesis, the present *in silico* results of our study suggest that monitoring the induction of specific miRs may provide the means to assess the antiaging and chemopreventive properties of particular dietary compounds.

## 1. Introduction

The DNA in each of our cells accumulates thousands of lesions every day. Cells are continuously challenged by DNA damage stimuli from various exogenous environmental factors, such as ultraviolet (UV) radiation, ionizing radiation (IR) and numerous chemical agents, or endogenous sources mainly represented by products of cellular metabolism. DNA damage can interfere with essential cellular processes, such as transcription or replication, and can compromise the viability of the cell. Specific DNA lesions can also induce mutations that cause cancer or other diseases, as well as contribute to the aging process [1]. DNA damage at the telomeric chromosomal sections definitely has pro-aging consequences [2]. Fortunately, cells have developed elaborate and efficient response pathways to preserve genomic stability. The DNA damage response (DDR) [3] is an evolutionarily-conserved signaling cascade activated by DNA damage (DD), which directs cell fate toward DNA repair, senescence or apoptosis [4]. DDR is a signaling network initiated by lesion recognition and amplified by multiple mediator signaling proteins, which eventually activate downstream effectors to modulate cell fate. Multiple DNA repair pathways have evolved to resolve various DNA lesions, including: base excision repair that removes damaged bases; mismatch repair that recognizes base incorporation errors and base damage; nucleotide excision repair that removes bulky DNA adducts; and cross-link repair that removes inter-strand cross-links. In addition, breaks in the DNA backbone are repaired via double-strand break (DSB) repair pathways, including homologous recombination (HR) and nonhomologous end joining [5]. If DNA lesions are not properly repaired or amended during replication, they can be converted into permanent mutations. When this occurs at the sites of oncogenes or tumor suppressor genes, the risk of neoplastic cell transformation is significantly increased [1,6]. In higher organisms, successful DDR is thought to prevent neoplastic transformation in a cell-autonomous manner, by ensuring removal of cells with severely damaged DNA [7]. DDR signaling has been suggested as a key mechanism linking DNA damage accumulation, cell senescence and organismal aging. In fact, during the cell lifetime, the genomic DNA is continuously exposed to different exogenous or endogenous factors that destabilize its integrity and functionality. Therefore, genomic instability and decline of DNA repair efficiency is considered one of the main drivers of the aging process [8]. However, emerging data suggest that DDR signaling can also work through a paracrine/systemic mechanism, shaping the systemic environment through the regulation of tissue repair and immune responses [9]. Given the fundamental role of DDR in maintaining genome integrity, this complex signaling network requires accurate regulatory mechanisms to respond to different types of DNA lesions in different stages of the cell cycle.

Recently, microRNAs (miRs) have emerged as important players in regulatory networks affecting the DNA damage/repair process in a wide range of physiological and pathological conditions [5,10]. miRs are 18–25 nucleotide non-coding RNAs that post-transcriptionally regulate gene expression stalling the translation of the cognate mRNA or promoting its degradation [11]. miRs have been identified to influence physiological processes, such as development, growth and differentiation [12], and have also been implicated in a wide range of diseases [13]. Multiple miRs may target the same miR, and the majority of miRs contain multiple binding sites for miRs, generating a highly complex regulatory network system by which hundreds of genes involved in different signaling pathways can be regulated simultaneously [14].

Nutrients and their bioactive compounds can modulate the miRs’ expression involved in many physiological and pathological processes [15]. Nutrition is the process that offers different substances to an organism that can work as energy suppliers (carbohydrate and fat), as cell structure sources (proteins) and on metabolism control (vitamins and minerals), thereby maintaining its homeostasis. The importance of diet and nutrition in human health and disease is well established. Basic laboratory research, clinical trials and epidemiological studies demonstrated that nutrient-rich bioactive foods can induce epigenetic changes and alter genes’ expression by the alteration of the histone structure, DNA methylation and miRs’ modulation [16].

Epi-gallocatechin-3-gallate (EGCG), resveratrol (RSV), curcumin (CRC) and n3-PUFA (n3-polyunsaturated fatty acids) are among the most studied compounds shown to have beneficial effects on human health [17,18]. EGCG, RSV and CRC are polyphenols present in fruits and vegetables. n3-PUFAs, are polyunsaturated fatty acids found in plants, as linolenic acid (ALA), or in fish eicosapentaenoic acid (EPA); and docosahexaenoic acid (DHA). Each of these four dietary compounds, at a concentration that potentially may be achievable in the organism, has been shown to suppress cell proliferation and induce apoptosis in certain types of human cancer cells [18,19]. In addition to potential anticancer activity, they possess cardiovascular protective properties [20,21] and beneficial effects on degenerative diseases [22]. Furthermore, an anti-inflammatory and antioxidant activity has been frequently associated with these compounds [23]. Most importantly, all of them may exert these effects by modulating miRs’ expressions [24,25]. In order to investigate and compare the impact of these specific food-derived compounds on DDR processes, we performed a literature search to identify miRs involved in DD/DDR and modulated by these dietary compounds. An *in silico* analysis using the DIANA software web-server was applied to identify targets and pathways that play a major role in the DD/DDR modulation by these compounds [26]. The results of the analysis of the pathways allowed us to speculate how food intervention could modulate DD/DDR.

## 2. Results

### 2.1. miRs Involved in DD/DDR and Bioactive Compounds Modulated

The results of a literature search for miRs involved in DD/DDR processes are reported in Table 1. Table 2 shows the literature search results for miRs modulated by each of the four compounds: EGCG, CRC, RSV and n3-PUFA, including the tissue/cell type, dose/concentration and duration of exposure of cells/tissue to the compound used in the cited study. Most of the studies we found with our search criteria (see Materials and Methods) have been performed *in vitro* and most of them on different human cancer cells.

**Figure 1:**
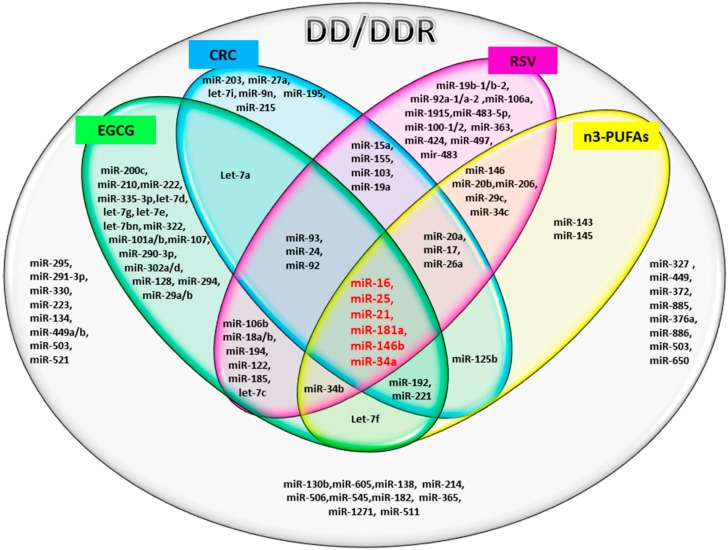
Venn diagram showing the microRNAs involved in DD/DDR (ellipse, grey) and identified as modulated by bioactive compounds: EGCG (epi-gallocatechin-3-gallate; green), CRC (curcumin; blue), RSV (resveratrol; pink) and n3-PUFAs (n3-polyunsaturated fatty acids, yellow). The common miRNAs, modulated by all four compounds, are indicated in red (miR-16, miR-25, miR-21, miR-181a, miR-146b, miR-34a).

The Venn diagram in Figure 1, shows the common and distinct miRs modulated by bioactive compounds and DD/DDR processes. The literature analysis indicates that a large number of the DD-associated miRs can be modified by dietary bioactive compounds. Furthermore, this analysis also revealed that the expression of some miRs seems to be compound class specific, while others miRs seem to be modulated by more than one bioactive compound. Interestingly, we found six miRs that were common to all of the compounds (indicated in red in Figure 1).

### 2.2. In Silico Analysis of Pathways Shared by Different miRs Involved in DD/DDR and Modulated by Compounds

**Figure 2:**
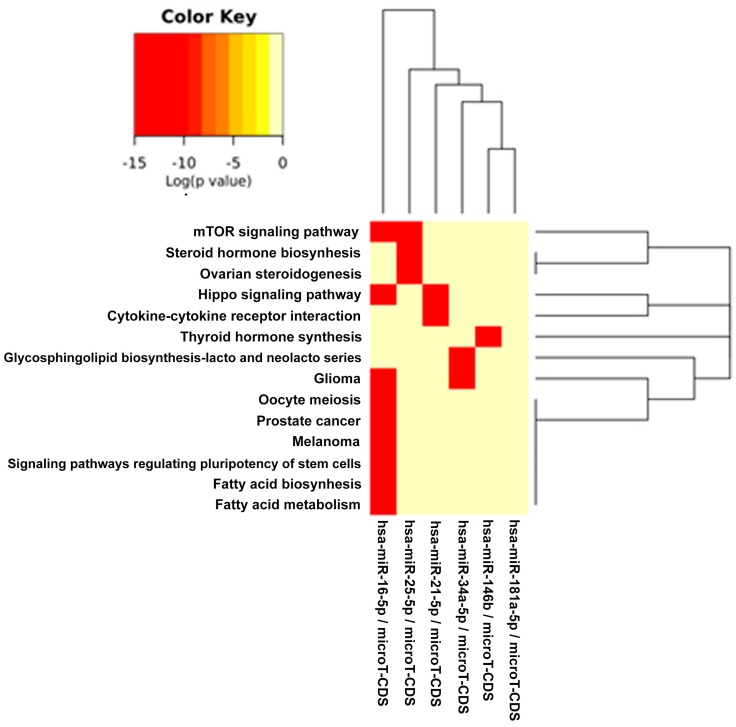
Binary heat map of pathways related to the common microRNAs involved in DDR signaling and modulated by all of the compounds: EGCG, CRC, RSV, n3-PUFAs. In this plot, heat map calculation is based on binary *p*-values (0: not targeted, 1: targeted); all significantly targeted pathways are marked with deep red. The plot shows miRNAs targeting similar pathways and pathways being targeted by miRNAs.

**Figure 3:**
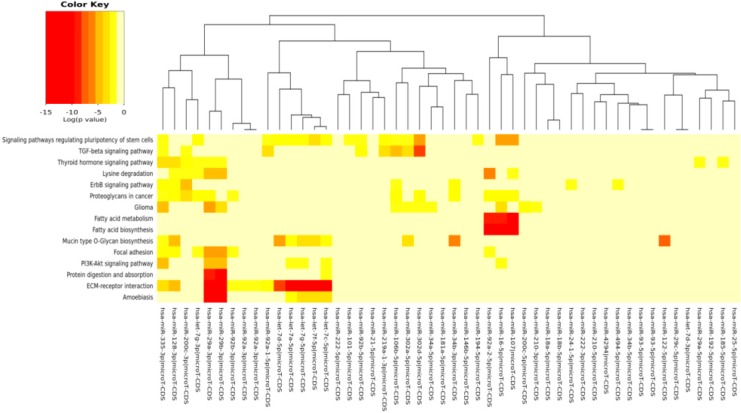
MicroRNAs involved in DDR and modulated by EGCG *versus* the pathways’ heat map. In this plot, heat map calculation is based on absolute *p*-values. Darker colors represent lower *p*-values (higher significance). The plot shows miRNAs targeting similar pathway clusters and pathways being targeted by miRNA groups.

**Figure 4:**
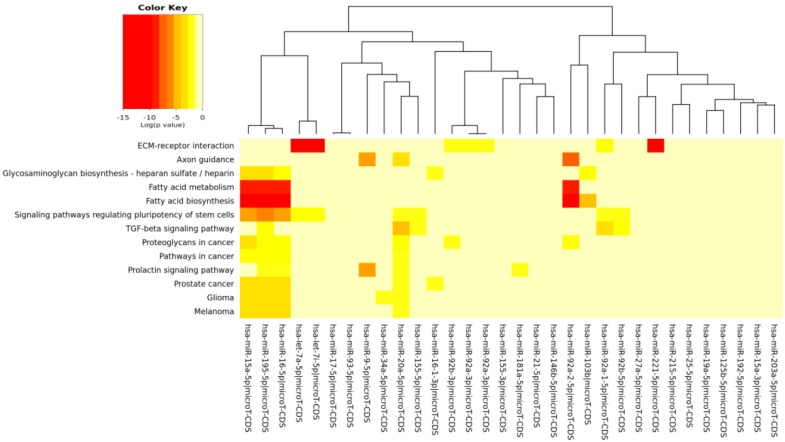
MicroRNAs involved in DDR and modulated by CRC *versus* the pathways’ heat map. In this plot, heat map calculation is based on absolute *p*-values. Darker colors represent lower *p*-values (higher significance). The plot shows microRNAs targeting similar pathway clusters and pathways being targeted by miRNA groups.

**Figure 5:**
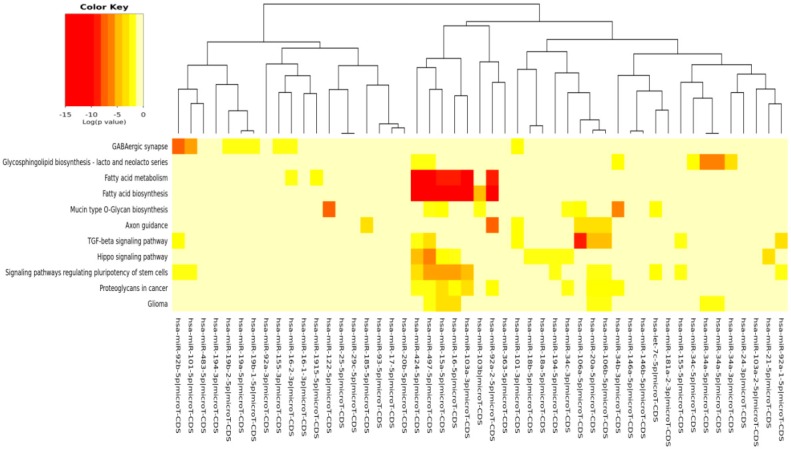
MicroRNAs involved in DDR and modulated by RSV *versus* the pathways’ heat map. In this plot, heat map calculation is based on absolute *p*-values. Darker colors represent lower *p*-values (higher significance). The plot shows microRNAs targeting similar pathway clusters and pathways being targeted by miRNA groups.

**Figure 6:**
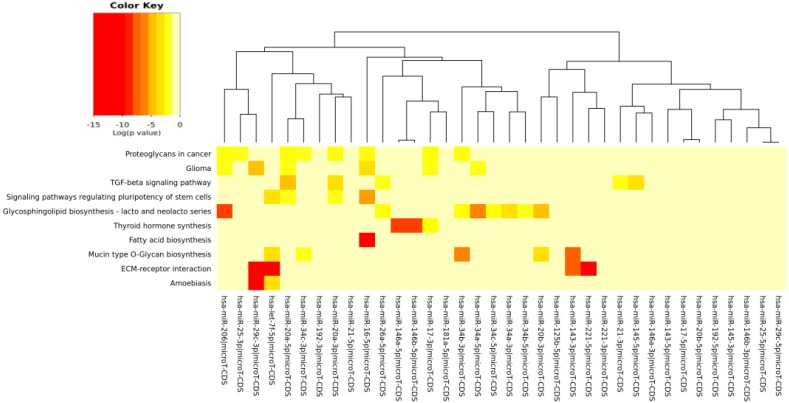
MicroRNAs involved in DDR and modulated by n3-PUFAs *versus* the pathways’ heat map. In this plot, heat map calculation is based on absolute *p*-values. Darker colors represent lower *p*-values (higher significance). The plot shows miRNAs targeting similar pathway clusters and pathways being targeted by miRNA groups.

For the *in silico* analyses reported in Table 3, Table 4, Table 5, Table 6 and Table 7 and Figure 2, Figure 3, Figure 4, Figure 5 and Figure 6, common miRNAs between Table 1 and Table 2 and reported in Venn diagram (Figure 1) were used.

Table 3 depicts the statistically-significant enriched KEGG pathways for miRs involved in DD/DDR and modulated by all compounds together (EGCG, CRC, RSV and n3-PUFA). The visual representation of the binary heat map showing the miRNAs/pathways interaction is reported in Figure 2. We found 14 KEGG pathways significantly related to genes targeted by miRs regulated by all four compounds. A consistent number of pathways were involved in energy metabolism, including the “mTOR signaling”, “fatty acid biosynthesis” and “fatty acid metabolism” pathways. Cancer-related pathways were also present, including glioma, melanoma and prostate cancer. In addition, we found significant pathways relevant in stem cell biology and tissue homeostasis, such as “signaling pathways regulating pluripotency of stem cells” and the “Hippo signaling pathway”.

The pathways significantly enriched (*p* < 0.05) for the target of miRs modulated by each specific compound (EGCG, CRC, RSV and n3-PUFA) are reported in Table 4, Table 5, Table 6 and Table 7, respectively. We found KEGG pathways, such as “fatty acid biosynthesis” and “signaling pathways regulating pluripotency of stem cells” significantly modulated by each compound. The visual representations of the heat maps showing the miR-pathway interaction, for each single compound, are reported in Figure 3, Figure 4, Figure 5 and Figure 6.

## 3. Discussion

The maintenance of genome integrity by an efficient DNA repair is of paramount importance in the prevention of cancer, attenuation of aging processes and age-related degenerative diseases. Effective DDR is the key mechanism providing genome stability. miRs play a critical role in the regulation of DDR. In this *in* *silico* study, therefore, we examined the influence of dietary bioactive compounds on the miRs involved in DDR, which potentially may offer clues on the role of these compounds as potential chemopreventive and antiaging (geroprotective) modalities.

The results of our analysis show a strong impact of the compounds considered on important miRs involved in DD/DDR pathways. In particular, the analysis suggests a synergic action of all compounds in the modulation of six miRs. Among these common miRs, we found miR-21 and miR-146. It has recently been highlighted that miR-146, miR-155 and miR-21 have a kay role in the interplay among DDR, cell senescence, inflammation and age-related diseases [9]. This trio of miRNAs, termed “inflamma-miRs”, has been primarily associated with chronic, low-grade inflammation known to characterize human aging and predisposing to age-related diseases [67]. We here find that two of these miRs, DD/DDR-associated (miR-146 and miR-21), are modulated by all four studied compounds and one of them, miR-155, by CRC and RSV [57]. miR-146 is one of the major miRs involved in orchestrating immune and inflammatory signaling via modulation of NF-kB activation [9]. Both miR-146a and miR-146b-5p have been found to target the DSB repair key protein BRCA1 [10].

miR-21 is a key modulator in many inflammatory pathways, and its aberrant expression in numerous cancers has led to its designation as an “onco-miR” [68]. miR-21 is induced by DD, negatively regulating G1/S transition. It also participates in the DNA damage-induced G2/M checkpoint [5]. It has been also shown that mR-21 negatively regulates Cdc25A and cell cycle progression in colon cancer cells [69]. In addition, miR-21 is upregulated during hepatitis C virus infection and negatively regulates IFN-α signaling through MyD88 and IRAK1; it may thus be a potential therapeutic target for antiviral intervention [70]. Recent observations suggest that miR-21 in cooperation with miR-145 (modulated by n3-PUFAs) play critical roles in the regulation of colon cancer stem cells [71]. miR-155 has been reported to regulate inflammation and immune responses [72]. miR-155 may have different functions in innate and adaptive immune responses, and the systemic diffusion of this DDR/-related miR may have either adverse or beneficial effects, depending on overall senescence/immunological host condition [9]. In DDR network, miR-155 modulates cell cycle after DD by targeting key genes involved in cell cycle control, such as WEE1 [73]. miR-155 has been shown to control the expression of TRF1, the protein that negatively regulates telomere length [74]. Indeed, the TRF1 gene is known to be a target of miR-155 [75]. Telomere length may also be regulated by miR-34a [76], which is in turn modulated by all four compounds, as demonstrated by our *in silico* analysis. miR-34b and miR-34c are also modulated by the three and two studied compounds, respectively. The miR-34 family, is a direct transcriptional target of p53, whose induction by DD and oncogenic stress pervades in diverse aspects of the DD response pathway [27]. Noteworthy, the compounds being evaluated here modulate members of the miR-17/92 cluster and miR-106a/b clusters (DDR modulated), having an important role in cancer and other numerous diseases [77]. In particular, our analysis reveals that RSV is able to modulate all of the miRs of these tree important clusters.

Our previous studies have demonstrated that RSV decreased the level of constitutive DD signaling by the reduced expression of γH2AX in proliferating A549, TK6 and WI-38 cells and in mitogenically-stimulated human lymphocytes [78]. The reduction of γ-H2AX was paralleled by a drop in the level of endogenous ROS and a decline in mTOR/S6K1 signaling [79]. H2AX is the initial sensor protein in the DSB response that can detect and mark DD by its phosphorylated form (γ-H2AX) [80,81]. H2AX is a target of miR-24 [80]. The present analysis suggests that RSV, as well as CRC and EGCG might regulate γH2AX expression by miR-24 mediation.

The DIANA program allowed us to investigate the common pathways associated with miRs. Among the most popular pathways involved in DD and DDR influenced by the compounds considered in this study, we found the fatty acids’ biosynthesis pathway and fatty acids’ metabolism. Alterations in fatty acid metabolism in cancer cells have received less attention, but are increasingly being recognized. Indeed, some studies have suggested that the DNA damage response is involved in the regulation of metabolic homeostasis. DNA damage could impair metabolic organ functions by causing cell death or senescence [82]. There is a strong relationship between DD and energy metabolism [83], and an implication for its role in tumorigenesis has been indicated. p53, the key factor in the DD/DDR network, has been reported to regulate fatty acid oxidation [84], and genes involved in fatty acid metabolism are regulated by p53 in different cell types and in response to both oncogenic stress and DD [85]. mTOR holds also an important role in lipid biosynthesis and metabolism [86,87] demonstrating a cross-talk among these pathways, and this fact has a significant meaning in cancerogenesis and chemoprevention. For example, by downregulating mTOR signaling and energy metabolism, it is possible to suppress the malignant phenotype of colorectal cancer cells [88,89]. Indeed, fatty acid synthase is a potential therapeutic target in cancer. There is strong evidence that constitutive mTOR signaling is the driving force of cellular and organismal aging and induction of senescence [90,91]. Extracellular matrix (ECM)-receptor interaction is also shown to be strongly involved in the DD/DDR and compounds interaction (EGCG, n3-PUFA, CRC). This is of interest in light of the evidence that ECM proteins are involved in cancer progression and outcome. The initiation of cell transformation is generally associated with genetic alterations in normal cells that lead to the loss of intercellular- and/or extracellular-matrix (ECM)-mediated cell adhesion [92]. There is a connection between ECM proteins, such integrin signaling and DNA repair [93]. There is also strong evidence that hyaluronic acid (HA; hyaluronate), the key component of ECM, by neutralizing the ROS, is reducing the induction of DNA damage by endogenous and exogenous oxidants [94,95,96]. Particularly interesting is the role of HA, which is a large constituent of stem cells’ niche, in protecting the integrity of genome of stem cells against reactive oxidants [95]. Our *in silico* results evidence that signaling regulating the pluripotency of stem cells pathway is clearly targeted by many miRs selected in this study, as evidenced by the heat maps of specific compounds. miRs represent an important layer of regulation for stem cell self-renewal and differentiation [97,98]. Among DDR/compound-modulated miRs, in our analysis, let-7, miR-302 and miR-17-92 have been found in previous studies as regulators of cellular pluripotency [99]. Signaling regulating the pluripotency of stem cells converges towards the activation of the transcriptional network and of many different pathways, which often show cross-talk in the determination of stem cell function. Indeed, FOXO, e.g., transcription factors, is required for DNA damage-induced growth arrest checkpoints [100], and as a further example, the mTOR pathway has been shown to be a major regulator of both ROS levels and autophagy in human stem cells [101]. The life-long persistence of stem cells in the body makes them particularly susceptible to the accumulation of cellular damage, which ultimately can lead to cell death, senescence or loss of regenerative function. Indeed, stem cells in many tissues have been found to undergo profound changes with age, exhibiting blunted responsiveness to tissue injury, dysregulation of proliferative activities and declining functional capacities. These changes translate into reduced effectiveness of cell replacement and tissue regeneration in aged organisms [102]. Noteworthy, miR-146a/b and miR-195, which we found modulated by all compounds and by CRC, respectively, have been reported as age-induced miRNAs involved in stem cell senescence [103]. These recent findings have demonstrated that silencing miR-195 reverses the senescence clock in aged stem cells by telomerase reactivation. The regulation of telomere length performed by the above-mentioned miR-155 may also have an important role in the cancer stem cell fate and cancer tissue homeostasis. miR-155 is upregulated in many cancers and can promote cancer stem cell phenotypes in liver [104]. RSV has been demonstrated to be able to decrease its levels, supporting our prediction analysis [57].

The availability of nutritional agents that can target specific miRs regulating stem cells’ function can improve regenerative potential in the body and, finally, counteract aging and prevent diseases, such as cancer.

It must be considered that most of the literature here analyzed refers to experimental research carried out on *in vitro* models. The results indicated that exposure time and concentrations of compounds can differently affect miRNA expression in different human cells. In addition another important issue evident in the literature [41] is the poor bioavailability of phytochemicals due to their metabolism, absorption and, consequently, the fact that most phytochemical compounds reach the targeted sites at sub-therapeutic concentrations [105]. These observations pave the way for *in vivo* dietary intervention studies and clinical trials focused on setting the appropriate dosage of the bioactive compounds that can effectively affect the miRs’ expression in humans.

The comparison of pathways identified, with our *in silico* analysis, to those identified experimentally in the study examples utilized here interestingly demonstrated that new pathways were evidenced from our prediction. Even more interesting, however, is that some of our predicted pathways were indeed found as directly implicated in some cancer studies and modulated by the compounds here considered.

The fact that experimental literature results have found that pathways, such as, for example, “fatty acid biosynthesis” and “fatty acid metabolism”, were actually affected by the compounds taken into account in this paper validated our prediction. This positive inter-comparison proves that the pathways computationally found by us were, indeed, the same found experimentally involved in some cancer models and in some senescence studies, confirming the validity of our prediction study. For examples, in breast and lung cancer cells, EGCG affected fatty acid metabolism [106,107]. Resveratrol was found to suppress cancer cell proliferation by inhibiting the fatty acid synthase signaling pathway [108]. Curcumin, as well, induces apoptosis, inhibiting intracellular fatty acid synthase in human breast cancer [109]. Clinical studies corroborate these findings [110], and trials were also carried out [111]. Resveratrol and EGCG are also found to improve the functional activity of the membrane lipids in aged liver models by influencing lipid composition and metabolism [112,113].

Presently, miRs have been already found to be modulated by the compounds here considered. On the other hand, other studies found that some of the pathways here evidenced are modulated by our compounds, but we provide here the link between miRs’ expression and the pathways’ modulation by the respective compounds. Thus, the importance of our results consists, indeed, of the possibility of detecting experimentally the link between miRs’ expression and pathways’ modulation.

Some authors suggested an interplay among miRNA, DD/DDR, specific signaling pathways and cancer promotion and invasion [114,115], and bioactive compounds play an important role in this interplay.

Taken together, the clinical relevance of such observations could be related to the bioactive compounds’ chemopreventive value in some type of tumors, as for example in breast cancers or lung cancers, where enough evidence corroborates this hypothesis.

In conclusion, since DDR and these pathways are strongly related to aging and carcinogenesis, the *in silico* results of our study suggest that monitoring the induction of specific miRs may provide the means to assess the antiaging and chemopreventive properties of particular bioactive compounds. Furthermore, the results of this *in silico* study indicate the most important pathways potentially modulated by dietary compounds and provide a framework for the generation of new strategies to design experimental studies and, finally, new anti-cancer and anti-aging therapies.

## 4. Materials and Methods

### 4.1. Search Strategy and Selection Criteria

A literature search was performed using PubMed databases to identify miRNAs with experimental evidence of involvement in DD and DDR (last accessed on 15 January 2016). The following keywords were used: “microRNA” and “DNA damage”. Due to the vast amount of literature, we selected in this paper the most significant and extensive reviews. We subsequently replaced “DNA damage” with “DNA damage response” or with “DNA damage repair” (see Table 1 in the Results).

Regarding miRs and the specific compounds considered in this paper, we did additional searches using “microRNA” and “epigallocatechin gallate” as key terms, and we substituted “epigallocatechin gallate” with “curcumin”, “resveratrol” or “n3-PUFA”. The literature search was limited to reports in English and in humans (see Table 2 in the Results).

The miRNAs involved in the DD/DDR network (from Table 1) and modulated by bioactive compounds (from Table 2) were selected to build the Venn diagram (Figure 1).

### 4.2. In Silico Analysis

All miRs used in the *in silico* analysis were selected as common between Table 1 and Table 2 and reported in the Venn diagram (Figure 1).

In Table 3 and Figure 2, the miRs found modulated by all four compounds were used (red in Figure 1). In Table 4, Table 5, Table 6 and Table 7 and in Figure 3, Figure 4, Figure 5 and Figure 6, the miRs modulated by a single compound were used.

To identify molecular pathways potentially altered by the expression of specific miRNAs selected from the literature search, the online software DIANA-mirPathv3 [26] was used. The software has the capacity to analyze the combinatorial effect of different miRNAs on Kyoto Encyclopedia of Genes and Genomes (KEGG) pathways. DIANA-mirPathv3 combines the gene targets of the selected miRs into a superset (union) performing the enrichment analysis and calculating the significance levels (*p*-values) between each miRNA and every pathway. The *p*-value is a measure of the association between a selected gene from the list of pathways. In pathway analysis, generally, *p*-values higher than 0.05 indicate that the association is not statistically significant, and the pathway could be rejected. In our analyses of pathways prediction, miR-gene interactions are derived from the *in silico* miRNA target prediction algorithms: DIANA-microT-CDS. The settings utilized were: *p*-value threshold of 0.05; DIANA-microT-CDS threshold of 0.85; merging method: pathways union. Fisher’s method was used to combine the results of more than one independent test bearing on the same hypothesis. All of the results were corrected in the combined enrichment analysis for multiple hypotheses testing, by applying Benjamini and Hochberg’s algorithm [116].

The results of the miR-pathway interaction are presented as “heat maps” (graphical representations of data where values in a matrix are represented as colors). They enable the visualization of a very large number of variables. The software DIANA-mirPathv3 utilizes the hierarchical clustering results on both axes (pathways and miRNAs), in order to construct the heat map visualization.

## Figures and Tables

**Table 1:** MicroRNAs involved in DD/DDR processes.

miRs	Reference
miR-34a/b/c, miR-192, miR-215, miR-16-1, miR-143, miR-107, let-7, miR-200c, miR-16, miR-145, miR-134, miR-449a/b, miR-503, miR-21, miR-24, miR-421, miR-504, miR-125b, miR-106b, miR-21, miR-210, miR-373, miR-100, miR-195, miR-124a, miR-290 cluster (miR-291-3p, miR-294, miR-295)	[5,27]
miR-363, miR-25, miR-542	[28]
miR-421, miR-24, miR-34a/b/c, miR-504, miR-125b, miR-302, miR-92, miR-192, miR-194, miR-215, miR-106a-92 cluster (miR-106a, miR-18b, miR-20b, miR-19b-2, miR-92a-2, miR-363), miR-106b/25 cluster (miR-106b, miR-25, miR-93), miR-210, miR-128, miR-20, miR-130b, miR-143, miR-145, miR-16-1, miR-16, miR-103, miR-26a, miR-206	[27]
miR-15a, miR-29, miR-107, miR-605, miR-17-92 cluster (miR-17, miR-18a, miR-19a, miR-20a, miR-19b-1, miR-92a-1), miR-21-605, miR-221, miR-222, miR-138, miR-223, miR-181a, miR-27a, miR-214, miR-101, miR-185, miR-100, miR-506, miR-545, miR-124, miR-9, miR-182, miR-146a	[10]

**Table 2:** MicroRNAs modulated by bioactive compounds; the effect on human normal or cancer cells.

Compound	miRNA	Reference	Cells/Cancer	Dose/Duration
EGCG	miR-18, miR-16, let-7a, miR-221, miR-34b, miR-193b, miR-222, miR-342	[29]	hepatic cancer	100 μM, 24 h
	miR-636, miR-3907	[30]	normal dermal fibroblasts	10 μM, 24 h
	miR-200c	[31]	colorectal cancer cells and colon cancer stem cells	100 μM, 24 h
	miR-210, miR-98-5p	[32,33]	lung cancer	40 μM, 9 h 10 μM, 24 h
	miR-1, miR-126	[34,35]	osteosarcoma	0.08 g/L, 48 h 0.2 g/L, 72 h
	miR-194	[36]	hepatocarcinoma	10 μg/mL, 48 h
	miR-7-1, miR-34a, miR-99a, miR-92, miR-93, miR-106b	[37,38]	neuroblastoma	50 μM, 24 h
	miR-25, miR-92, miR-141, miR-200a	[39]	Hela cells and lymphoblasts	1–5 μM, 24 h
	miR-33a, miR-122	[40]	hepatocarcinoma	50 μM, 1 h
EGCG	miR-92, miR-93, miR-106b, miR-7-1, miR-34a, miR-99a	[41]	neuroblastoma	50 μM, 24 h
	miR-467bn, miR-487b, miR-197, miR-805, miR-374n, let-7f, miR-350, miR-24-1n, miR-137, miR-335-3p, let-7a, miR-222, miR-26b, miR-30c-1n, let-7d, miR-98, miR-30c, miR-30bn, miR-32, miR-674n, miR-532-5p, let-7g, miR-18a, miR-192, miR-302d, miR-30b, miR-802, let-7e, miR-322, miR-720, miR-146b, miR-340-3p, miR-185, miR-425, miR-10a, miR-126-5p, miR-101a, miR-30en, let-7c, miR-141, miR-33, miR-29an, miR-199b, miR-450a-5p, miR-21, miR-23a, miR-101b, miR-148a, miR-193, miR-23b, miR-107, miR-140, miR-551b, miR-466c-5p, miR-106a, miR-590-3p, miR-875-3p, miR-224, miR-292-5p, miR-678, miR-469, let-7bn, miR-463n, miR-574-3p, miR-201, miR-290-3p, miR-181a, miR-302a, miR-429, miR-133a, miR-190b, miR-710, miR-135b, miR-296-5p, miR-191n, miR-188-5p, miR-298, miR-181a-1n, miR-466g, miR-26bn, miR-466f-3p, miR-29bn, miR-1224, miR-291b-5p, miR-324-5p, miR-486, miR-128, miR-450b-3p, miR-135an, miR-294, miR-671-5p, miR-878-3p, miR-801, miR-370, miR-1, miR-494, miR-133b	[41]	hepatocarcinoma	100 μM, 24 h
CRC	miR-192-5p/215	[42]	lung cancer	15 μM, 48 h
	miR-7	[43]	pancreatic cancer	3–6 μM, 72 h
	miR-22	[44,45]	retinoblastoma	20 μM, 48 h
	miR-27a	[46]	colon cancer	2.5–10 μg/mL, 24 h
	miR-21	[47]		
	let-7a, miR-21, miR-34a	[48]	esophageal cancer	30 μM, 24 h
	miR-221	[44,49]	pancreatic cancer	500 nM of synthetic CRC analogue, 72 h
CRC	miR-27a, miR-20a, miR-17-5p, miR-21	[41]	colon carcinoma	30 μM, 24 h
	miR-203	[41]	bladder carcinoma	10 μM, 3 days
	miR-320, miR-26a, let-7i, miR-130a, miR-16, miR-125b, miR-23a, miR-27b, miR-155, miR-625, miR-576-3p, miR-186n, miR-9n, let-7i	[50]	lung adenocarcinoma	15 μM, 48 h
	miR-15a, miR-16-1	[41]	leukemic cells	5–20 μM, 24-72 h
	miR15a, miR-16	[51]	breast cancer	10–60 μM, 24 h
	miR-103, miR-140, miR-146b, miR-148a, miR-15b, miR-181a, miR-181b, miR-181d, miR-195, miR-196a, miR-199an, miR-19a, miR-204, miR-20a, miR-21, miR-22, miR-23a, miR-23b, miR-24, miR-25, miR-26a, miR-27a, miR-34a, miR-374, miR-510, miR-7, miR-92, miR-93, miR-98	[41]	pancreatic cancer	10 μM, 72 h
RSV	miR-663, miR-744m	[44,52]	breast cancer	100 μM, 24 h
	miR-21	[44,53]	pancreatic cancer	50 μM, 24 h
	miR-520h	[54]	lung cancer	10–20 μM, 48 h
	miR-21, miR-181b, miR-663, miR-30c2	[55]	peripheral blood mononuclear cells from hypertensive patients	RSV (8 mg) grape extract, one year daily intake ( *in vivo* study)
	miR-150, miR-296-5p	[56]	lymph node cancer prostate	50 μM, 24 h
	miR-33a, miR-122	[40]	hepatocarcinoma	50 μM, 1 h
	miR-155 miR-663	[57]	monocytic cells	30–50 μM, 14 h
RSV	miR-155, miR-34a	[58]	EBV-immortalized B cells	25–50 μM, 24 h
	miR-7, miR-17, miR-18b, miR-20a, miR-20b, miR-92b, miR-106a, miR106b, miR-17-5p, miR-20a, miR-106b, miR-17-92 cluster, miR-106ab clusters	[59]	prostate cancer	50–100 μM, 24 h
	miR-622	[41]	bronchial epithelial cells	50 μM, 48 h
	miR-155, miR-633	[41]	monocytes	30 μM, 14 h
	let-7c, miR-106a, miR-106b, miR-1224-5p, miR-1228, miR-231, miR-1246, miR-1260, miR-1267, miR-1268, miR-129, miR-1290, miR-1308, miR-1469, miR-149, miR-150, miR-152, miR-15a, miR-17, miR-1825, miR-185, miR-18b, miR-1908, miR-1915, miR-197, miR-1972, miR-1973, miR-1974, miR-1975, miR-1977, miR-1979, miR-20a, miR-20b, miR-24, miR-296-5p, miR-483-5p, miR-513a-5p, miR-548q, miR-572, miR-575, miR-612, miR-638, miR-654-5p, miR-659, miR-671-5p, miR-7, miR-762, miR-764, miR-874, miR-92b, miR-939	[41]	lymph node cancer prostate	50 μM, 48 h
	miR-1, miR-100-1/2, miR-102, miR-103-1, miR-103-2, miR-146a, miR-146b-5p, miR-16-0, miR-17, miR-181a2, miR-194-2, miR-196a1, miR-205, miR-206, miR-21, miR-23a, miR-23b, miR-25, miR-26a, miR-29c, miR-30a-3p, miR-30c-1, miR-30d, miR-30e-5p, miR-323, miR-340, miR-363n-5p, miR-424, miR-494, miR-497, miR-560, miR-560, miR-565, miR-565, miR-572, miR-574, miR-594, miR-615, miR-622, miR-629, miR-631, miR-638, miR-639, miR-657, miR-659, miR-663, miR-801, miR-92a-2	[41]	colorectal carcinoma	50 μM, 14 h
n3-PUFA	miR-192, miR-30c, miR-141-3p, miR-221-3p, miR-1283, let-7f, miR-181a-5p, miR-1, miR-30a	[60]	Caco-2 cells	200 μM DHA in lipid micelles, 24 h
n3-PUFA	miR-26a, miR-26b	[61]	cholangiocarcinoma	50 μM DHA, 12 h
	miR-221	[62]	endothelial progenitor cells	25–125 μM EPA, 4 h
	miR-146, miR-181a	[63]	glioma	25–50 μM DHA, 48 h
	miR-21	[64]	breast cancer	152 nM DHA, 24 h
	miR-30c, miR-20b, miR-16, miR-22, miR-145, miR-34, miR-25, miR-17, miR-26a, miR-29c, miR-200a, miR-206, miR-323, miR-16, miR-22, miR-20b, miR-30c, miR-183, miR-224, miR-145, miR-181a, miR-208, miR-143, miR-20a, miR-149, miR-125b	[19,65]	glioma	50–100 μM DHA, 24 h

**Table 3:** Enriched KEGG pathways for predicted targets of the DD/DDR microRNAs modulated by all four compounds.

KEGG Pathway	*p*-Value	#of Genes	miRNAs
Fatty acid biosynthesis	0	4	miR-16
Fatty acid metabolism	2.23 × 10^-5^	7	miR-16
Thyroid hormone synthesis	2.58 × 10^-5^	5	miR-146b
Signaling pathways regulating pluripotency of stem cells	0.0002346931	24	miR-16
Glioma	0.000556152	15	miR-16 miR-34a
Glycosphingolipid biosynthesis: lacto and neolacto series	0.001440903	2	miR-34a
Hippo signaling pathway	0.00410767	24	miR-16 miR-21
Steroid hormone biosynthesis	0.009497578	1	miR-25
Ovarian steroidogenesis	0.01398717	1	miR-25
Melanoma	0.01891852	14	miR-16
Prostate cancer	0.02681897	16	miR-16
Cytokine-cytokine receptor interaction	0.03260184	11	miR-21
mTOR signaling pathway	0.03417768	13	miR-16 miR-25
Oocyte meiosis	0.03995201	16	miR-16

DIANA-miRPath v3.0 [66] was used to predict the targeted KEGG pathways by miRs involved in DD/DDR signaling and modulated by all considered compounds (EGCG, CRC, RSV and n3-PUFAs). The target prediction threshold was set at 0.85. *p*-value < 0.05. ^#^: number.

**Table 4:** Enriched KEGG pathways for predicted targets of the DD**/**DDR microRNAs modulated by EGCG.

KEGG Pathway	*p*-Value	# of Genes	# of miRNAs
Fatty acid biosynthesis	0	4	3
ECM-receptor interaction	0	42	13
Signaling pathways regulating pluripotency of stem cells	2.44 × 10^−9^	76	17
Amebiasis	3.53 × 10^−6^	24	6
Proteoglycans in cancer	1.17 × 10^−4^	117	12
Mucin type *O*-glycan biosynthesis	1.67 × 10^−2^	15	10
Glioma	1.43 × 10^−1^	39	10
TGF-β signaling pathway	1.85 × 10^0^	40	8
Fatty acid metabolism	2.28 × 10^1^	9	3
Focal adhesion	0.0002057474	110	7
PI3K-Akt signaling pathway	0.001829865	138	7
Lysine degradation	0.006232265	18	7
ErbB signaling pathway	0.02315742	45	7
Protein digestion and absorption	0.02773474	26	3
Thyroid hormone signaling pathway	0.03049808	57	8
Glycosaminoglycan biosynthesis heparan sulfate/heparin	0.05012798	9	5

DIANA-miRPath v3.0 was used to predict the targeted KEGG pathways by miRs involved in DDR signaling and that were EGCG modulated. The target prediction threshold was set at 0.85. *p*-value < 0.05. ^#^: number.

**Table 5:** Enriched KEGG pathways for predicted targets of the DDR microRNAs modulated by CRC.

KEGG Pathway	*p*-Value	# of Genes	# of miRNAs
Fatty acid biosynthesis	0	4	5
ECM-receptor interaction	0	19	7
Fatty acid metabolism	6.88 × 10^−9^	8	4
Signaling pathways regulating pluripotency of stem cells	2.16 × 10^−5^	55	9
Glioma	0.0002249224	20	5
Proteoglycans in cancer	0.0003169881	66	6
TGF-β signaling pathway	0.006985672	28	5
Prostate cancer	0.009151789	31	5
Axon guidance	0.01011865	45	3
Melanoma	0.01304415	20	4
Prolactin signaling pathway	0.01811186	28	5
Pathways in cancer	0.0206275	70	4
Glycosaminoglycan biosynthesis-heparan sulfate/heparin	0.02616968	3	5

DIANA-miRPath v3.0 was used to predict the targeted KEGG pathways by miRs involved in DDR signaling and that were CRC modulated. The target prediction threshold was set at 0.85. *p*-value < 0.05. ^#^: number.

**Table 6:** Enriched KEGG pathways for predicted targets of the DDR microRNAs modulated by RSV.

KEGG Pathway	*p*-Value	# of Genes	# of miRNAs
Fatty acid biosynthesis	0	5	7
Fatty acid metabolism	0	16	8
Signaling pathways regulating pluripotency of stem cells	1.07 × 10^−6^	64	13
TGF-β signaling pathway	1.39 × 10^−4^	34	9
Proteoglycans in cancer	8.1 × 10^−1^	70	10
Axon guidance	0.0009836851	55	6
Hippo signaling pathway	0.001005551	43	9
Mucin type *O*-glycan biosynthesis	0.002219981	12	8
Glycosphingolipid biosynthesis: lacto and neolacto series	0.006851962	6	7
GABAergic synapse	0.007317003	13	8
Glioma	0.03097475	21	7

DIANA-miRPath v3.0 was used to predict the targeted KEGG pathways by miRs involved in DDR signaling and that were RSV modulated. The target prediction threshold was set at 0.85. *p*-value < 0.05. ^#^: number.

**Table 7:** Enriched KEGG pathways for predicted DDR targets of the microRNAs modulated by n-3-PUFAs.

KEGG Pathway	*p*-Value	# of Genes	# of miRNAs
ECM-receptor interaction	0	27	4
Fatty acid biosynthesis	5.57 × 10^-5^	4	1
Glycosphingolipid biosynthesis: lacto and neolacto series	2.21 × 10^-2^	8	8
Mucin type *O*-glycan biosynthesis	0.0003249162	9	5
Proteoglycans in cancer	0.0004576113	76	8
TGF-β signaling pathway	0.001807704	27	5
Thyroid hormone synthesis	0.002952912	5	3
Amebiasis	0.00834241	24	2
Signaling pathways regulating pluripotency of stem cells	0.02548584	51	4
Glioma	0.02578235	31	6

DIANA-miRPath v3.0 was used to predict the targeted KEGG pathways by miRs involved in DDR signaling and that were n3-PUFAs modulated. The target prediction threshold was set at 0.85. *p*-value < 0.05. ^#^: number.

## Abbreviations

EGCG	Epi-gallocatechin-3-gallate
RSV	Resveratrol
CRC	Curcumin
n3-PUFAs	n3-polyunsaturated fatty acids
miR	micro-RNA
DD	DNA damage
DDR	DNA damage response
